# Supplementary material for: Machine learning based estimation of field-scale daily, high resolution, multi-depth soil moisture for the Western and Midwestern United States
Source: PeerJ. 2022 Nov 4;10:e14275. doi: 10.7717/peerj.14275 (PMC9639422; doi:10.7717/peerj.14275)
Supplement: Supplemental Information 2 [file peerj-10-14275-s002.docx]

**Table S1:**

**Broad-scale soil moisture datasets available for the contiguous U.S. that are generated with data fusion using remote sensing imagery and environmental covariates.**

| **Spatial** | | **Temporal** | | **Max depth** | **Soil moisture database ^a^** | **Covariates ^b^** | **Model ^c^** | **Validation scheme and result ^d^** | **Reference** |
| --- | --- | --- | --- | --- | --- | --- | --- | --- | --- |
| **Extent** | **Scale** | **Coverage** | **resolution** |  |  |  |  |  |  |
| Global | 50 m | 2014-2020 | Vary with location and data availability | 5 cm | Sentinel-1, GLDAS, Landsat 8, in-situ SM | soil texture, BD, EVI, SWIR, LULC, SRG | GBRT | Random independent set: R^2^ (0.81); RMSE (0.04m^3^/m^3^) | (Greifeneder et al., 2021) |
| Global | 100 m | 2016-2019 | Every 3 days | 5 cm | SMAP, Sentinel-1, in-situ SM | soil texture, SOC, BD, LULC, DEM, SRG | QRF | Independent sites: R^2^ (0.30-0.53);  RMSE (0.05-0.08 m^3^/m^3^) | (Huang et al., 2020) |
| Global | 2.25 km | 2015-2017 | Every 2 to 3 days | 5 cm | SMAP, in-situ SM | NDVI, TI | ANN | Independent sites: R^2^ (~0.6);  RMSE (~0.065 m^3^/m^3^) | (Alemohammad et al., 2018) |
| Global | 0.05° | 2001-2012 | Monthly | 5 cm | ECV (CCI), in-situ SM | ALB, NDVI, LST, LULC, DEM | RF | Independent sites: R^2^ (0.66) | (Jing et al., 2018) |
| Global | 0.25° | 2000-2018 | Daily | 5 cm | CCI, in-situ SM | soil texture, ppt, NDVI, LST, EVI | RF | Random independent set: R^2^ (0.81); RMSE (0.05 m^3^/m^3^) | (Zhang et al., 2021) |
| Global | 0.25° | 2000-2019 | Daily | 50 cm | GLDAS, in-situ SM | soil porosity, soil and vegetation type, air T, ppt, HMD, SR, LST, EL | LSTM | Independent sites: R^2^ (~0.45); | (O. & Orth, 2021) |
| North America | 1 km | After 2002 **^e^** | Daily | Surface | AMSR-E | ALB, LST, VI | GWR, MW, LR | Comparison between original and downscaled: R^2^ (0.70-0.74) | (Yu et al., 2008) |
| U.S. | 30 m | 2015-2019 | Every 3 h 30 mins | Surface | SMAP, in-situ SM | Soil texture, soil hydraulic properties, ppt, ALB, VOD, SWR, LULC, SRG, DEM | LSM, RTM, RF | Independent sites: R^2^ (~0.4-0.6);  RMSE (~0.06-0.08 m^3^/m^3^) | (Vergopolan et al., 2021) |
| U.S. | 400 m or 1 km | 2018-2019 | Every 8 days | Surface | SMAP, NLDAS, in-situ SM | ppt, NDVI, LST, LAI | ATIM | Independent sites: R^2^ (0.34-0.49);  RMSE (0.068-0.082 m^3^/m^3^); | (Fang et al., 2021) |
| U.S. | 1 km | 2015 | Daily | 5 cm | SMAP, in-situ SM | soil texture, ppt, NDVI, LST, DEM | RF | Independent sites: R^2^ (0.41) | (Abbaszadeh et al., 2019) |
| U.S. | 1 km | 2015-2018 | Daily | Surface | SMAP, NLDAS, in-situ SM | ppt, NDVI, VNIR, LST | ATIM | Independent sites: R^2^ (0.54) | (Fang et al., 2020) |
| U.S. | 1 km | Real-time | Daily | 5 cm | SMAP, Sentinel-3, in-situ SM | EVI, LST | DISPATCH | Independent sites: R^2^ (0.41) | (Yin et al., 2020) |
| U.S. | 1 km | 1991-2016 | Annual | 5 cm | CCI, NASMD | DEM | DTA | Independent sites: R^2^ (0.46);  RMSE (5.7 m^3^/m^3^); | (Guevara & Vargas, 2019) |
| U.S. | 1 km | 2015-2019 | Daily | 100 cm | SMAP, in-situ SM | Soil texture, BD, ppt, NDVI, EVI, GPP, LST, DEM | XGB | Independent sites: R^2^ (0.50 for 5cm, 0.47 for 10cm, 0.42 for 20cm, 0.27 for 50cm, and 0.15 for 100cm) | (Karthikeyan & Mishra, 2021) |
| U.S. | 3 km | 2015-2016 | Every 3 days | 5 cm | SMAP, NLDAS, in-situ SM | air T, pet, EL | DISPATCH | Independent sites: R^2^ (0.22) | (Mishra et al., 2018) |
| U.S. | 9 km | 2015-2018 | Daily | 5 cm | SMAP, NLDAS, in-situ SM | Soil texture, BD, ALB, LULC, NDVI | LSTM | Random independent set: R^2^ (0.81); RMSE (0.034 m^3^/m^3^) | (Liu et al., 2022) |

^a^ AMSR2 = Advanced Microwave Scanning Radiometer 2 (Kachi et al., 2008); AMSR-E = Advanced Microwave Scanning Radiometer for EOS (Njoku et al., 2003); ECV (CCI) = Essential Climate Variables (Climate Change Initiative) soil moisture product (Dorigo et al., 2017); in-situ SM = in-situ soil moisture measured from field sensors; GLDAS = Global Land Data Assimilation System (Rodell et al., 2004); Landsat-8 = NASA and USGS’s Landsat 8 mission (Roy et al., 2014); NASMD = North American Soil Moisture Dataset; NLDAS = North American Land Data Assimilation System (Xia et al., 2012); Sentinel-1 = European Space Agency’s Sentinel-1 mission (Paloscia et al., 2013); SMAP = Soil Moisture Active Passive mission (Entekhabi et al., 2010).

^b^ air T = air temperature; ALB = albedo; BD = bulk density; BT = brightness temperature; DEM = digital elevation model; EL = elevation; EVI = Enhanced Vegetation Index; HMD = humidity; LAI = Leaf Area Index; LST = Land Surface Temperature; LULC = Land use land cover; NDVI = Normalized Difference Vegetation Index; pet = potential evapotranspiration; ppt = precipitation; SR = solar radiation; SRG = surface roughness; SWIR = shortwave infrared; SWR = shortwave radiation; TI = topographic index; VI = Vegetation Index; VNIR = visible and near infrared; VOD = vegetation optical depth.

^c^ ANN = Artificial Neural Network; ATIM = Adapted thermal inertia model; DISPATCH = DISaggregation based on Physical And Theoretical scale CHange; DTA = Digital terrain analysis; GBRT = Gradient Boosted Regression Trees; GWR = Geographically Weighted Regression; LR = linear regression; LSM = land surface model; LSTM = Long-short term memory model; MW = moving window; QRF = Quantile Random Forest; RF = Random Forest; RTM = radiative transfer model; XGB = Extreme Gradient Boosting algorithm.

^d^ Comparison between original and downscaled = correlation was calculated based on the original dataset used for downscaling and the downscaled dataset; Independent sites = study sites randomly divided into calibration and validation sites with results reported on the validation sites that are independent of the calibration sites; Random independent set = dataset randomly divided into calibration and validation with results reported on the validation set, while data points from the validation set could come from the same study site.

^e^ The main goal of the study was to test different algorithms rather than provide a readily available data product for the whole spatial coverage, therefore the temporal coverage is not applicable.

**Table S2:**

**A list of open-source codes provided by this work. The number of step corresponds to the codes deposited for the step.**

| **Function** | **Step** | **Platform ^a^** | **Description ^b^** | **Optional** |
| --- | --- | --- | --- | --- |
| Processing in-situ dataset for calibration and validation | 1 | R | Download, process, and quality-control SCAN datasets | N |
|  | 2 | R | Download, process, and quality-control USCRN datasets | N |
|  | 3 | GEE | Extract LULC classes from NLCD for retained SCAN and USCRN stations in order to refine the dataset further based on LULC classes of interests | Y |
|  | 4 | R | Combine quality-controlled SCAN and USCRN datasets processed from steps 1 and 2; Prepare lists needed for further extraction of site, site-year, site-date, and site-depth-based environmental covariates | N |
| Covariate extraction and quality control | 5 | Colab | Download and process multi-depth NLDAS dataset from hourly to daily; Export processed data as image collections | N |
|  | 6 | GEE | Extract soil, terrain, and LULC (including tree cover%) based on lists of site-depth, site, and site-year combinations, respectively | N |
|  | 7 | Colab | Extract climate and biotic covariates based on a list of site-date combinations | N |
|  | 8 | Colab | Extract NLDAS data based on site-date combinations for multiple depth layers | N |
|  | 9 | Colab | Combine all extracted biotic and climate covariates and NLDAS values from subsets | N |
|  | 10 | R | Merge all extracted covariates and soil moisture datasets | N |
|  | 11 | GEE | Extract RAP-based LULC% for stations from the Western U.S. | Y |
|  | 12 | R | Merge extracted RAP covariates with other covariates and soil moisture records | Y |
|  | 13 | Colab | Extract quality control flags for remote sensing covariates based on bitmasks | Y |
|  | 14 | Colab | Combine all extracted quality control flags for remote sensing covariates from subsets | Y |
|  | 15 | R | Refine datasets based on quality control flags generated for remote sensing covariates | Y |
| Model building, evaluation, and comparison | 16 | R | Build and save tree-based model for the quality-controlled full dataset | N |
|  | 17 | R | Build and save tree-based model for the quality-controlled regionalized (Western US) dataset that includes RAP covariates | Y |
|  | 18 | R | Build tree-based model for each site using the quality-controlled full dataset | Y |
|  | 19 | R | Build tree-based model for different soil depth, LULC, and sampling time classes using the quality-controlled full dataset | Y |
|  | 20 | R | Build tree-based model using the quality-controlled full dataset spiked with local samples for grassland | Y |
| Model application/ prediction | 21 | GEE | Generate temporally constant or year-based site covariates | N |
|  | 22 | Colab | Generate temporally dynamic site covariates | N |
|  | 23 | R | Predict soil moisture using the tree-based model derived from quality-controlled regionalized dataset | N |
|  | 24 | Colab | Upload predicted moisture data layer to GEE | Y |
|  | 25 | GEE | Data visualization at the point and site level for quality checking | Y |

^a^ GEE = Google Earth Engine (JavaScript-based language); Colab = Google Colaboratory (Python-based language).

^b^ LULC = land use and land cover type; NLCD = National Land Cover Database; NLDAS = North American Land Data Assimilation System; RAP = Rangeland Analysis Platform; SCAN = Soil Climate Analysis Network; USCRN = U.S. Climate Reference Network.

**Table S3:**

**Pearson Correlation Coefficient (r) calculated between observed soil moisture and modeling covariates for the whole dataset and for the datasets divided by sampling depth, land cover classes, and month of the year.**

| **Groups** | **LSM ^a^** | **Soil ^b^** | | | | **RS ^c^** | | | | | **Climate ^d^** | | | **DEM ^e^** | | | | | | | |
| --- | --- | --- | --- | --- | --- | --- | --- | --- | --- | --- | --- | --- | --- | --- | --- | --- | --- | --- | --- | --- | --- |
|  | **SM** | **Clay** | **Sand** | **BD** | **SOC** | **LST** | **GPP** | **EVI** | **NDWI** | **Tree** | **ppt** | **TA** | **VPD** | **EL** | **SL** | **AS** | **TWI** | **mcurv** | **hcurv** | **vcurv** | **sr** |
| **All** | 0.55 | 0.46 | -0.55 | 0.10 | -0.08 | -0.25 | 0.15 | 0.05 | 0.06 | 0.13 | 0.07 | -0.10 | 0.18 | -0.43 | -0.07 | 0.03 | -0.10 | 0.05 | 0.05 | 0.03 | -0.12 |
| **Depth group** | | | | | | | | | | | | | | | | | | | | | |
| 5 cm | 0.70 | 0.20 | -0.45 | 0.06 | 0.09 | -0.43 | 0.11 | -0.04 | 0.03 | 0.15 | 0.08 | -0.24 | 0.17 | -0.49 | -0.02 | 0.05 | -0.14 | 0.03 | 0.02 | 0.04 | -0.07 |
| 10 cm | 0.62 | 0.35 | -0.54 | 0.10 | 0.03 | -0.34 | 0.11 | 0.01 | 0.07 | 0.07 | 0.07 | -0.19 | 0.14 | -0.43 | -0.07 | 0.03 | -0.15 | 0.05 | 0.06 | 0.03 | -0.12 |
| 20 cm | 0.59 | 0.44 | -0.62 | 0.05 | 0.13 | -0.26 | 0.14 | 0.04 | 0.06 | 0.09 | 0.07 | -0.12 | 0.17 | -0.43 | -0.06 | 0.04 | -0.10 | 0.06 | 0.09 | 0.01 | -0.11 |
| 50 cm | 0.49 | 0.53 | -0.64 | -0.07 | 0.07 | -0.12 | 0.18 | 0.12 | 0.04 | 0.16 | 0.07 | 0.01 | 0.23 | -0.45 | -0.10 | 0.01 | -0.05 | 0.08 | 0.08 | 0.05 | -0.16 |
| 100 cm | 0.44 | 0.58 | -0.59 | -0.07 | 0.09 | -0.12 | 0.23 | 0.17 | 0.07 | 0.23 | 0.07 | 0.02 | 0.24 | -0.45 | -0.07 | 0.01 | -0.09 | 0.05 | 0.05 | 0.02 | -0.12 |
| **LULC group** | | | | | | | | | | | | | | | | | | | | | |
| Grass | 0.50 | 0.42 | -0.53 | 0.02 | 0.01 | -0.22 | 0.16 | 0.07 | 0.14 | 0.11 | 0.05 | -0.09 | 0.09 | -0.32 | -0.16 | 0.34 | -0.18 | 0.23 | 0.20 | 0.16 | -0.20 |
| Crop | 0.42 | 0.38 | -0.49 | 0.07 | -0.03 | -0.21 | 0.01 | -0.05 | -0.04 | -0.01 | 0.07 | -0.08 | 0.19 | -0.48 | 0.32 | -0.20 | -0.06 | -0.16 | -0.02 | -0.25 | -0.03 |
| Forest | 0.65 | 0.49 | -0.57 | 0.47 | -0.41 | -0.19 | 0.02 | -0.07 | -0.15 | 0.18 | 0.08 | -0.15 | 0.09 | -0.48 | -0.24 | -0.01 | 0.17 | -0.05 | 0.05 | -0.10 | -0.39 |
| Pasture | 0.67 | 0.58 | -0.54 | 0.08 | -0.32 | -0.13 | -0.02 | -0.02 | -0.08 | 0.14 | 0.04 | -0.01 | 0.15 | -0.45 | -0.02 | 0.21 | -0.14 | -0.29 | -0.32 | -0.15 | -0.08 |
| Shrubs | 0.43 | 0.40 | -0.38 | 0.02 | -0.09 | -0.18 | 0.11 | 0.03 | 0.11 | -0.01 | 0.02 | -0.15 | 0.03 | -0.03 | -0.08 | 0.14 | -0.09 | 0.05 | 0.02 | 0.08 | -0.06 |
| **Month group** | | | | | | | | | | | | | | | | | | | | | |
| 1 | 0.45 | 0.43 | -0.62 | -0.06 | 0.09 | -0.15 | -0.07 | 0.01 | -0.07 | 0.26 | 0.02 | -0.11 | 0.09 | -0.51 | -0.01 | -0.01 | -0.06 | 0.04 | 0.05 | 0.02 | -0.03 |
| 2 | 0.47 | 0.43 | -0.64 | -0.06 | 0.13 | -0.23 | -0.01 | -0.01 | 0.01 | 0.25 | 0.05 | -0.16 | 0.15 | -0.50 | -0.01 | 0.01 | -0.10 | 0.09 | 0.07 | 0.07 | -0.02 |
| 3 | 0.54 | 0.42 | -0.64 | -0.05 | 0.14 | -0.25 | 0.09 | 0.02 | -0.04 | 0.27 | 0.06 | -0.06 | 0.30 | -0.40 | 0.04 | 0.04 | -0.14 | 0.13 | 0.11 | 0.11 | 0.03 |
| 4 | 0.60 | 0.45 | -0.64 | -0.01 | 0.08 | -0.35 | 0.17 | 0.02 | -0.01 | 0.22 | 0.08 | -0.08 | 0.32 | -0.40 | 0.01 | 0.02 | -0.13 | 0.08 | 0.06 | 0.07 | -0.02 |
| 5 | 0.62 | 0.46 | -0.60 | 0.05 | -0.01 | -0.34 | 0.25 | 0.07 | 0.07 | 0.15 | 0.10 | -0.01 | 0.38 | -0.40 | -0.03 | 0.01 | -0.12 | 0.06 | 0.05 | 0.05 | -0.07 |
| 6 | 0.61 | 0.49 | -0.55 | 0.14 | -0.14 | -0.33 | 0.32 | 0.18 | 0.21 | 0.08 | 0.11 | 0.06 | 0.41 | -0.44 | -0.09 | 0.01 | -0.11 | 0.03 | 0.03 | 0.02 | -0.16 |
| 7 | 0.51 | 0.50 | -0.51 | 0.17 | -0.19 | -0.31 | 0.32 | 0.29 | 0.29 | 0.04 | 0.09 | 0.13 | 0.40 | -0.40 | -0.12 | 0.04 | -0.08 | 0.04 | 0.05 | 0.02 | -0.18 |
| 8 | 0.46 | 0.50 | -0.51 | 0.18 | -0.19 | -0.31 | 0.33 | 0.26 | 0.28 | 0.04 | 0.11 | 0.11 | 0.36 | -0.40 | -0.12 | 0.05 | -0.09 | 0.05 | 0.06 | 0.02 | -0.19 |
| 9 | 0.49 | 0.49 | -0.52 | 0.16 | -0.15 | -0.28 | 0.32 | 0.18 | 0.15 | 0.08 | 0.07 | 0.10 | 0.36 | -0.45 | -0.11 | 0.04 | -0.10 | 0.03 | 0.04 | 0.01 | -0.17 |
| 10 | 0.53 | 0.45 | -0.55 | 0.10 | -0.07 | -0.22 | 0.21 | 0.08 | 0.01 | 0.14 | 0.05 | 0.01 | 0.21 | -0.45 | -0.09 | 0.05 | -0.09 | 0.10 | 0.03 | -0.01 | -0.15 |
| 11 | 0.57 | 0.43 | -0.61 | 0.05 | 0.03 | -0.22 | 0.10 | 0.02 | -0.05 | 0.24 | 0.02 | -0.07 | 0.15 | -0.55 | -0.07 | -0.01 | -0.08 | 0.03 | 0.03 | 0.02 | -0.10 |
| 12 | 0.52 | 0.42 | -0.64 | -0.03 | 0.10 | -0.15 | -0.03 | -0.04 | -0.05 | 0.27 | 0.04 | -0.09 | 0.12 | -0.56 | -0.03 | 0.03 | -0.07 | 0.06 | 0.08 | 0.03 | -0.06 |

^a^ Land surface model (LSM) covariate: SM = NLDAS-derived soil moisture.

^b^ Soil covariates: Clay = clay contents; Sand = sand contents; BD = bulk density; SOC = soil organic C.

^c^ Remote sensing (RS) covariates: LST = land surface temperature; GPP = gross primary productivity; EVI = enhanced vegetation index; NDWI = normalized difference wetness index; Tree = tree cover percentage.

^d^ Climate covariates: ppt = precipitation; TA = air temperature; VPD = vapor pressure deficit.

^e^ Digital Elevation Model (DEM) covariates: EL = elevation; SL = slope; AS = aspect; TWI = topographic wetness index; mcurv = mean curvature; hcurv = horizontal curvature; vcurv = vertical curvature; sr = surface roughness.

**Table S4:**

**Comparison of model fits including Coefficient of Determination (R^2^), Root Mean Square Error (RMSE), Mean Bias Error (MBE), and Residual Prediction Deviation (RPD) using reduced covariate datasets.**

| **Model ^a^** | **Covariate groups ^b^ and covariates ^c^** | **R^2^** | **RMSE (m^3^/ m^3^)** | **\|MBE\| (m^3^/ m^3^)** | **RPD** |
| --- | --- | --- | --- | --- | --- |
| R1 | NLDAS + Depth + LULC + Soils + RS + Climate | 0.47 | 0.090 | 0.007 | 1.36 |
| R2 | NLDAS + Depth + LULC + Soils + RS + DEM | 0.51 | 0.085 | 0.002 | 1.43 |
| R3 | NLDAS + Depth + LULC + Soils + DEM + Climate | 0.51 | 0.085 | 0.003 | 1.43 |
| R4 | NLDAS + Depth + LULC + RS + DEM + Climate | 0.41 | 0.096 | 0.020 | 1.27 |
| R5 | NLDAS + Depth + LULC + Soils + RS | 0.46 | 0.092 | 0.008 | 1.33 |
| R6 | NLDAS + Depth + LULC + Soils + Climate | 0.47 | 0.091 | 0.008 | 1.35 |
| R7 | NLDAS + Depth + LULC + Soils + DEM | 0.51 | 0.086 | 0.003 | 1.42 |
| R8 | NLDAS + Depth + LULC + RS + Climate | 0.34 | 0.105 | 0.025 | 1.17 |
| R9 | NLDAS + Depth + LULC + RS + DEM | 0.42 | 0.095 | 0.019 | 1.28 |
| R10 | NLDAS + Depth + LULC + Climate + DEM | 0.42 | 0.095 | 0.013 | 1.29 |
| R11 | NLDAS + Depth + LULC + Soils | 0.47 | 0.090 | 0.006 | 1.36 |
| R12 | NLDAS + Depth + LULC + RS | 0.33 | 0.107 | 0.024 | 1.14 |
| R13 | NLDAS + Depth + LULC + Climate | 0.35 | 0.103 | 0.022 | 1.18 |
| R14 | NLDAS + Depth + LULC + DEM | 0.42 | 0.095 | 0.015 | 1.29 |
| R15 | Sand + NLDAS + Clay + EL + BD + Depth + SOC + GPP + TWI + Tree + LULC + LST + sr + hcurv + VPD | 0.52 | 0.085 | 0.005 | 1.43 |
| R16 | Sand + NLDAS + Clay + EL + BD | 0.47 | 0.089 | 0.006 | 1.37 |

^a^ Reduced models 1-4 (R1-R4) = reduced models containing 5 covariate groups; Reduced models 5-10 (R5-R10) = reduced models containing 4 covariate groups; Reduced models 11-14 (R11-R14) = reduced models containing 3 covariate groups; Reduced model 15 (R15) = reduced model containing the top 15 most influential covariates; Reduced model 16 (R16) = reduced model containing the top 5 most influential covariates.

^b^ NLDAS = NLDAS-derived soil moisture; Depth = soil sampling depth; Soils = soil covariates; RS = Remote sensing covariates; Climate = climate covariates; DEM = Digital Elevation Model covariates.

^c^ Sand = sand contents; Clay = clay contents; EL = elevation; BD = bulk density; SOC = soil organic C; GPP = gross primary productivity; TWI = topographic wetness index; Tree = tree cover percentage; LULC = land use land cover class; LST = land surface temperature; sr = surface roughness; hcurv = horizontal curvature; VPD = vapor pressure deficit.

**Table S5:**

**Pearson Correlation for temporal trend between modeled and sensor measured soil moisture for different sensor locations at the Central Plains Experimental Range (CPER) site.**

| **Sensor** | **Total records** | **Percentage of records (%) from different depth layers (cm)** | | | | | **Correlation coefficient (r)** |
| --- | --- | --- | --- | --- | --- | --- | --- |
|  |  | **7** | **16** | **26** | **46** | **66** |  |
| 1 | 823 | 31 | 0 | 0 | 17 | 52 | 0.57 |
| 2 | 2019 | 14 | 30.5 | 0 | 30.5 | 25 | 0.39 |
| 3 | 1819 | 19 | 34 | 34 | 0 | 13 | 0.34 |
| 4 | 2167 | 17 | 28.5 | 0 | 28.5 | 26 | 0.51 |
| 5 | 1443 | 24 | 10 | 0 | 28 | 38 | 0.28 |

**Table S6:**

**Time and resources required to complete model prediction for multi-depth soil moisture at the test rangeland sites in Colorado. The estimation is based on generating soil moisture estimates for a whole year with the assumption that the scripts are run one at a time. The time required for building calibration models is not included since those steps do not need to be repeated by users.**

| **Step** | **Cedaredge ranch**  **(~ 10 km^2^)** | | **Central Plains Experimental Range (CPER) site**  **(~ 100 km^2^)** | | **Computing platform** |
| --- | --- | --- | --- | --- | --- |
|  | **Time** | **Total storage** | **Time** | **Total storage** |  |
| Extract 30 m soil and topographic covariates | < 1 min | ~ 1.5 MB | < 1 min | ~ 8 MB | Google Earth Engine |
| Extract daily 30 m climate and biotic covariates | ~ 40 mins | ~ 120 MB | ~ 1.5 hours | ~ 350 MB | Google Colaboratory |
| Extract daily NLDAS layers ^a^ | ~ 25 mins | ~ 2.5 MB | ~ 45 mins | ~ 7 MB | Google Colaboratory |
| Generate daily soil moisture estimates | ~ 3 hours 45 mins | ~ 20 MB | ~ 6 hours 40 mins | ~ 100 MB | R virtual machine on Google Cloud Computation Platform |
| Upload generated layers for visualization ^b^ | ~ 15 mins | NA ^b^ | ~ 35 mins | NA ^c^ | Google Colaboratory and Google Earth Engine |

^a^ NLDAS = North American Land Data Assimilation System.

^b^ This is an optional step where generated soil moisture layers are uploaded from Google Cloud bucket storage to Google Earth Engine using Google Colaboratory scripts.

^c^ This step does not use additional storage in Google Earth Engine because the images are directly read as Cloud-optimized assets from the Cloud bucket storage.


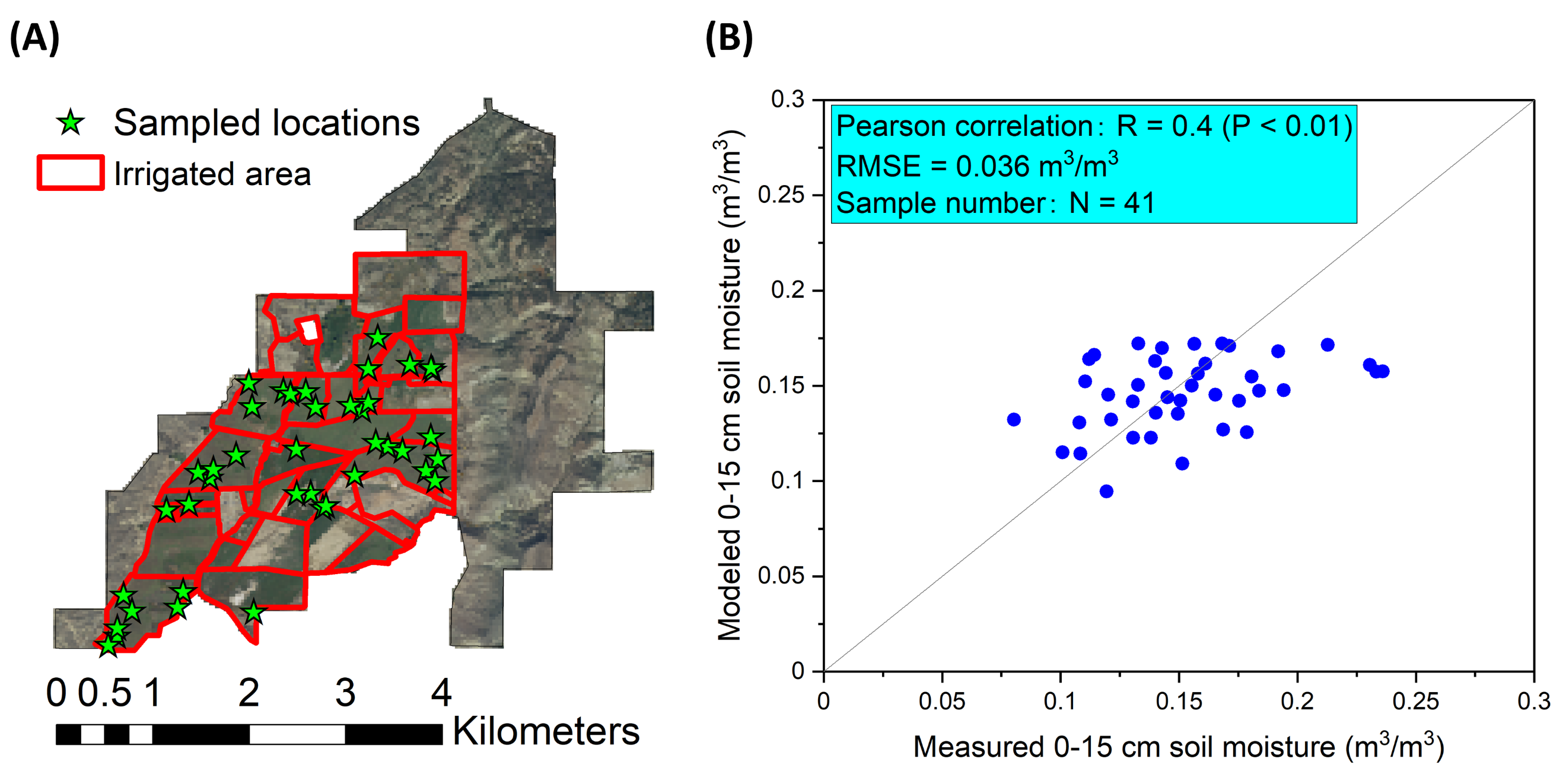


Figure S1. Validation of soil moisture estimates in spatial at the Cedaredge ranch site using gravimetric measurements carried out from (A) sampling locations within the irrigated area and shown as (B) the difference between measured and modeled soil moisture for the top 0-15 cm depth layer. The site in (A) is mapped upon the National Agriculture Imagery Program (NAIP) imagery.


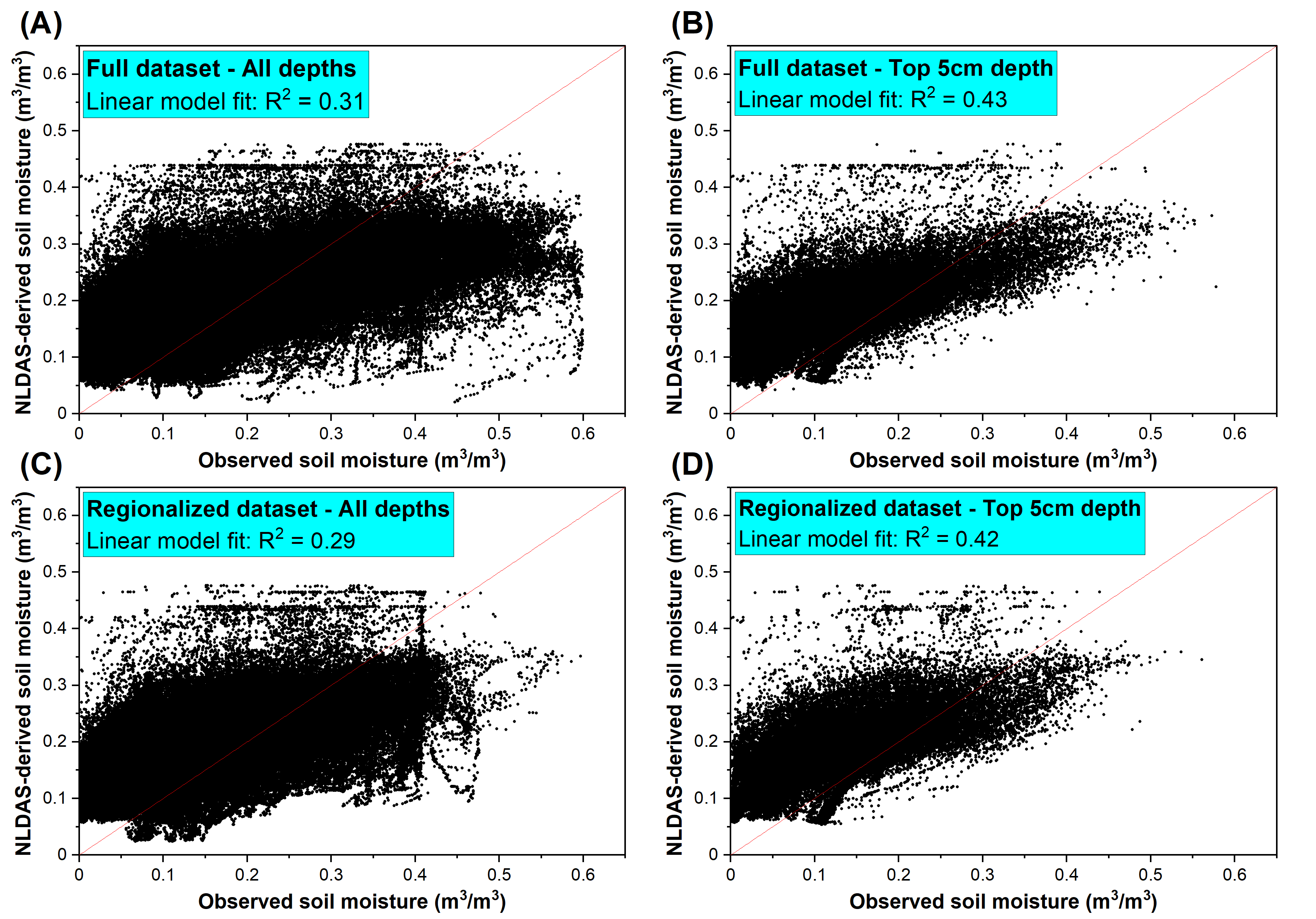


Figure S2. Comparison between field measured and North American Land Data Assimilation System (NLDAS)-derived soil moisture of the independent validation sites for (A) all depths and (B) surface 5 cm depth samples of the full dataset and for (C) all depths and (D) surface 5 cm depth samples of the regionalized dataset. The full dataset contains observations from the Midwestern and Western U.S. states while the regionalized dataset only contains observations from the Western U.S. states due to the use of covariates from the rangeland analysis platform data layers.


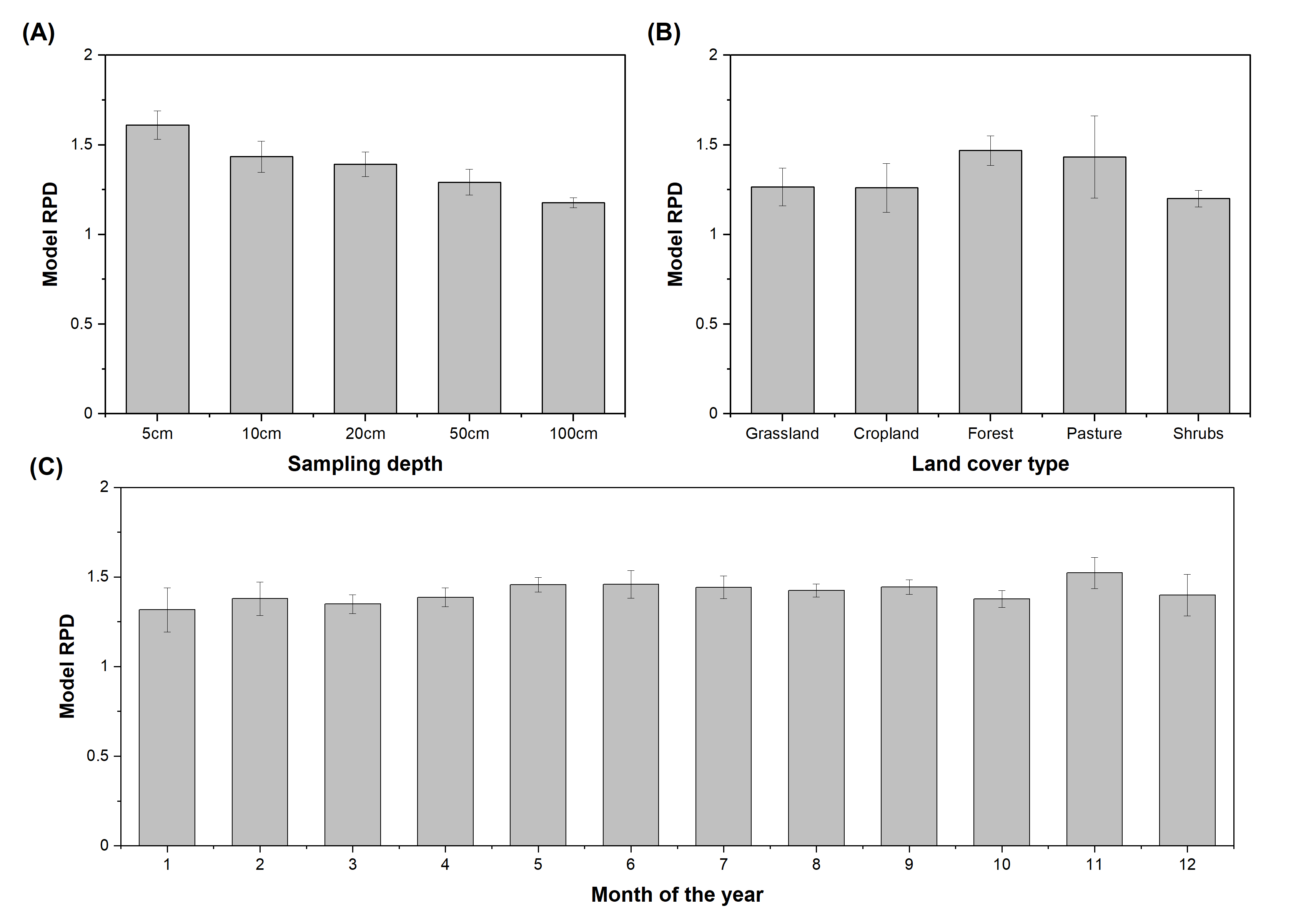


Figure S3. The model Residual Prediction Deviation (RPD) derived from independent validation for soil moisture models built for different (A) sampling depths, (B) land cover types, and (C) sampling months. Model performance is shown for soil sampling depth up to 100 cm for (B) and (C). The full dataset containing observations from the Midwestern and Western U.S. states was used to build the calibration models. The model performance was presented as mean $\pm$ standard deviation based on 5 model runs.


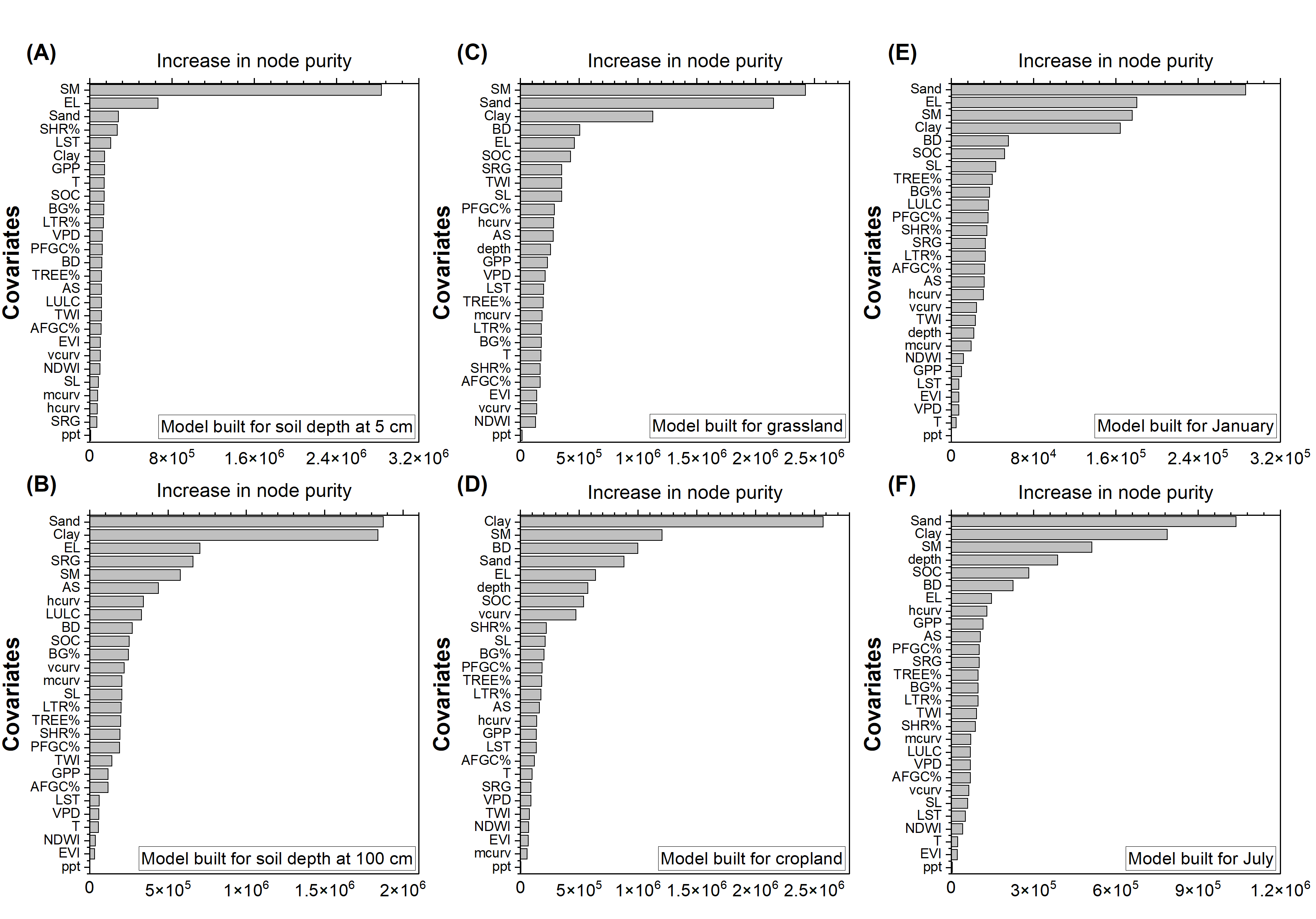


Figure S4. Variable importance ranked for soil moisture model built on soils from (A) 5 cm sampling depth, (B) 100 cm sampling depth, (C) grassland, (D) cropland, (E) January, and (F) July. The regionalized calibration dataset containing soil moisture observations from the Western U.S. states was used to build the models and the variable importance was reported based on the increase in node purity of the Quantile Random Forest model of the calibration dataset. The covariates include soil sand (Sand) and clay (Clay) contents, soil bulk density (BD), soil organic carbon (SOC), North American Land Data Assimilation System (NLDAS)-derived soil moisture (SM), precipitation (ppt), temperature (T), vapor pressure deficit (VPD), Gross Primary Productivity (GPP), Enhanced Vegetation Index (EVI), land use land cover (LULC), Land Surface Temperature (LST), Normalized Difference wetness Index (NDWI), elevation (EL), slope (SL), aspect (AS), mean (mcurv), vertical (vcurv), and horizontal (hcurv) curvatures, Topographic wetness Index (TWI), surface roughness (SRG), and estimates of annual herbs% (AFGC%), perennial herbs (PFGC%), bare ground (BG%), tree (TREE%), litter (LTR%), and shrub (SHB%) covers derived from the rangeland analysis platform (RAP) data layers.


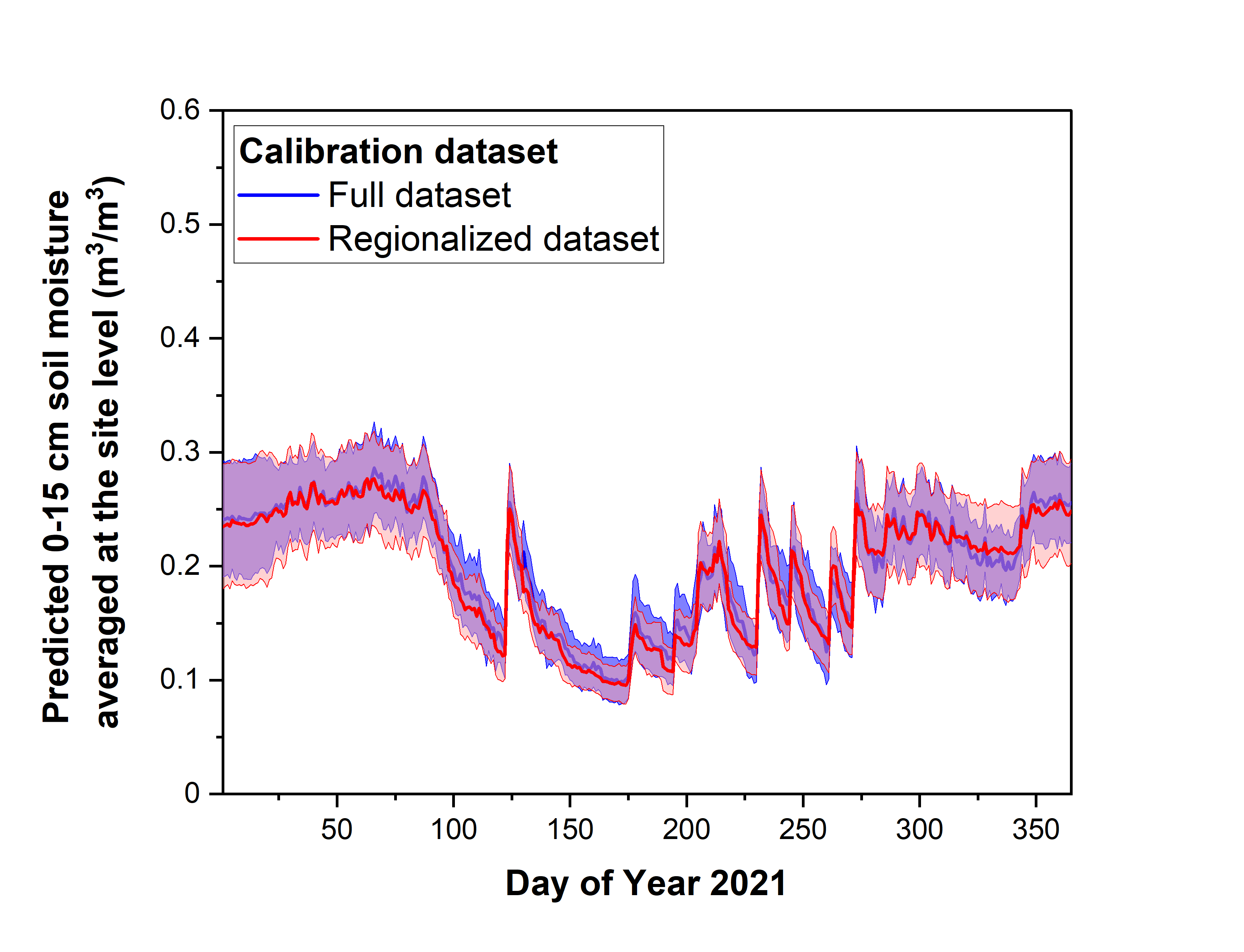


Figure S5. Temporal pattern of soil moisture predicted for the year 2021 for the 0-15 cm depth layers at based on different calibration datasets. The result is presented as mean$\pm$standard deviation based on the area of the Colorado Cedaredge ranch test site. The full dataset contains observations from the Midwestern and Western U.S. states while the regionalized dataset only contains observations from the Western U.S. states due to the use of covariates from the rangeland analysis platform (RAP) data product.


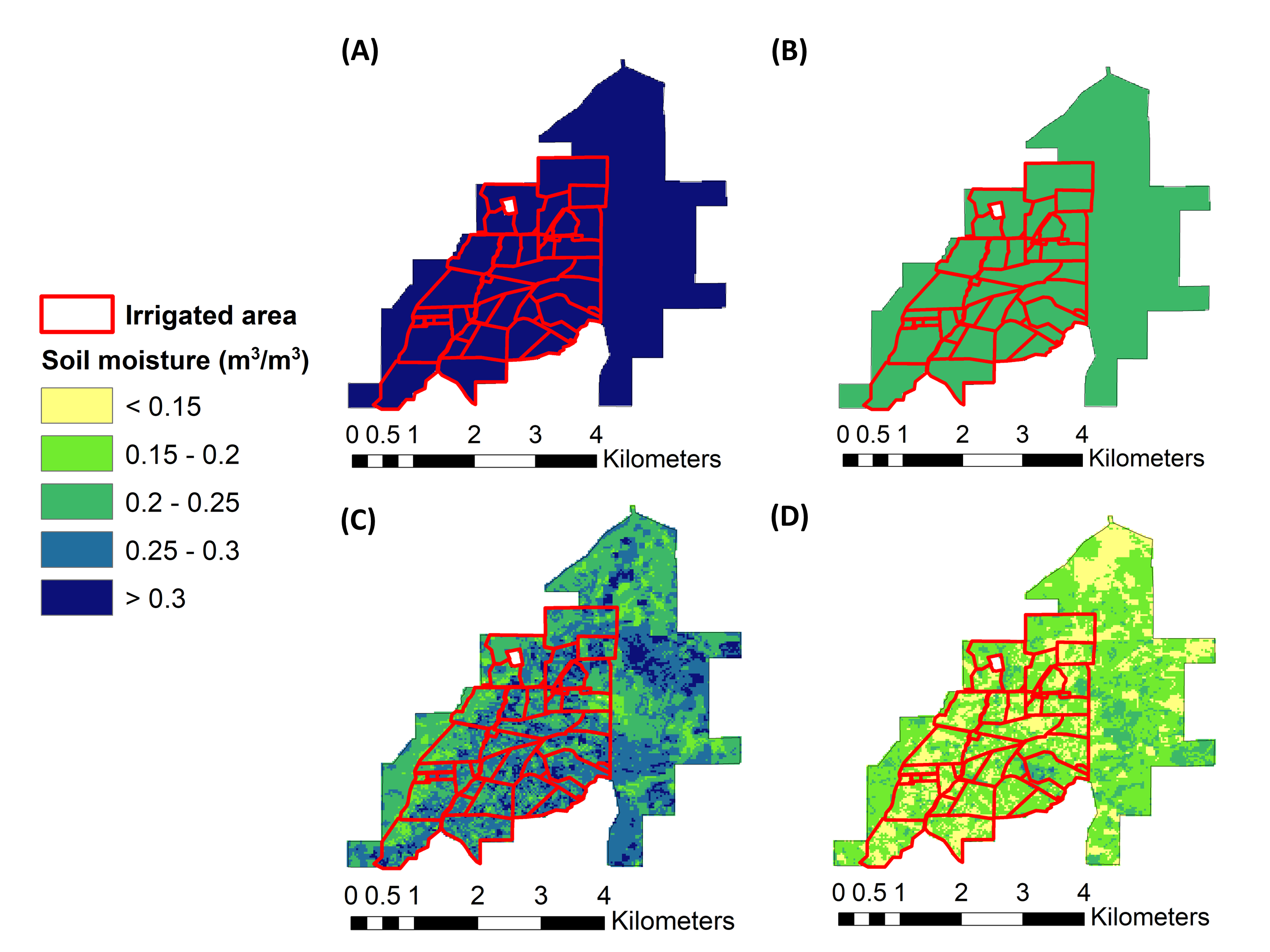


Figure S6. Soil moisture from 0-15 cm depth for the Colorado Cedaredge test ranch based on North American Land Data Assimilation System (NLDAS) for (A) a winter day (2019/01/01) and a summer day (2019/07/01) compared to model estimates generated at a spatial resolution of 30 m for the same (C) winter day and (D) summer day. The irrigated pasture portion of the ranch is marked with red lines in the map.


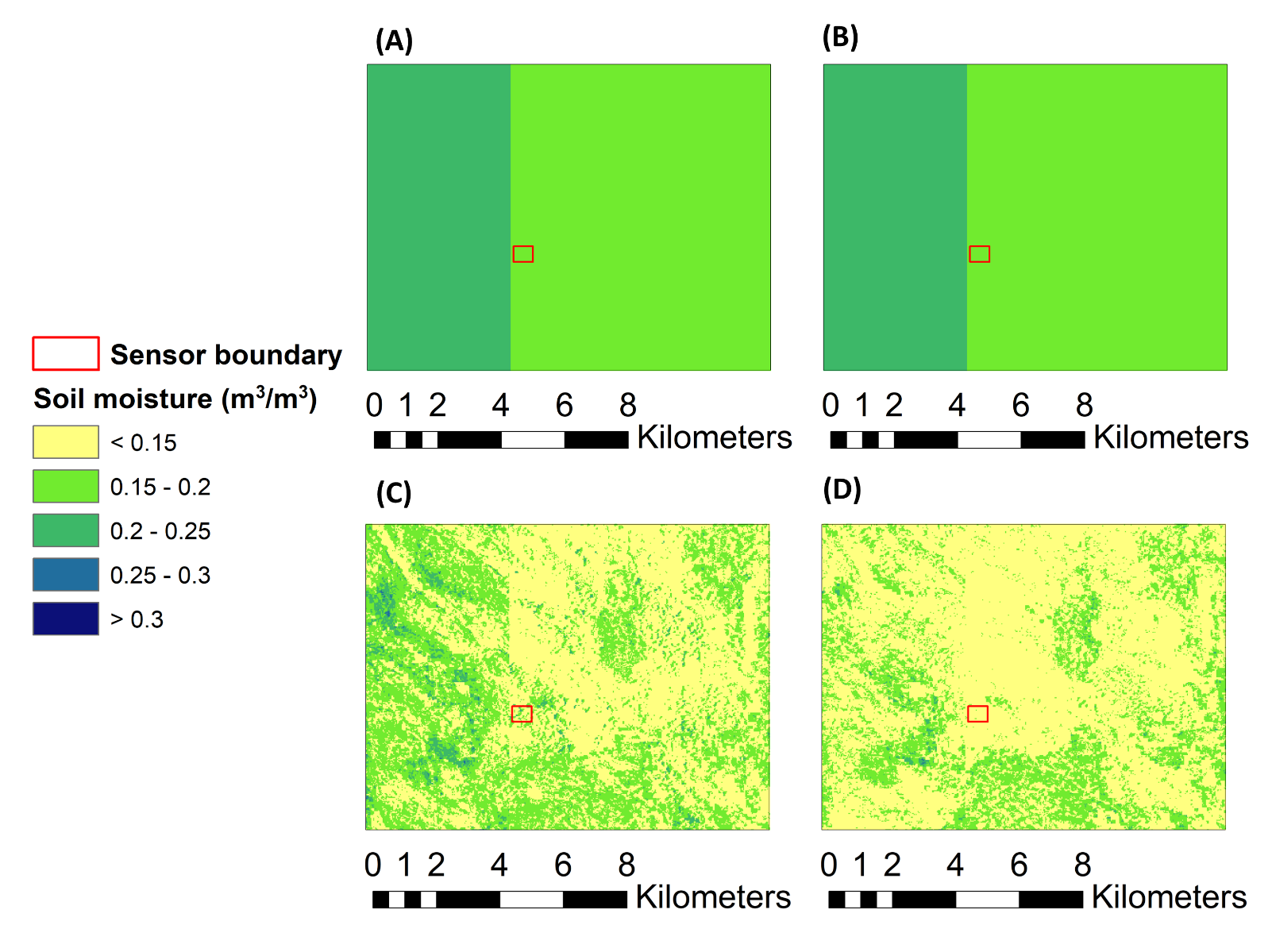


Figure S7. Soil moisture from 0-15 cm depth for the Central Plains Experimental Range (CPER) based on North American Land Data Assimilation System (NLDAS) for (A) a winter day (2019/01/01) and a summer day (2019/07/01) compared to model estimates generated at a spatial resolution of 30 m for the same (C) winter day and (D) summer day. The boundary for moisture sensors of the site is marked with red lines in the map.
